# The role of ferroptosis in chronic intermittent hypoxia-induced lung injury

**DOI:** 10.1186/s12890-022-02262-x

**Published:** 2022-12-27

**Authors:** Jia Chen, Huixin Zhu, Qin Chen, Yisong Yang, Mengxue Chen, Jiefeng Huang, Menglan Chen, Ningfang Lian

**Affiliations:** 1 Department of Respiratory and Critical Care Medicine, The First Affiliated Hospital of Fujian Medical University, No. 20, Chazhong Road, Taijiang District, Fuzhou, 350005 Fujian Province People’s Republic of China; 2 Fujian Provincial Sleep-Disordered Breathing Clinic Center, Institute of Respiratory Disease, Fujian Medical University, Fuzhou, Fujian People’s Republic of China; 3 Department of Respiratory and Critical Care Medicine, National Regional Medical Center, Binhai Campus of the First Affiliated Hospital, Fujian Medical University, Fuzhou, 350212 People’s Republic of China; 4 Department of Surgical Care Center, The First Affiliated Hospital of Fujian Medical University, Fuzhou, Fujian People’s Republic of China; 5 Clinical Skills Teaching Center, Fujian University of Traditional Chinese Medicine, Fuzhou, Fujian Province People’s Republic of China

**Keywords:** Obstructive sleep apnea, Chronic intermittent hypoxia, Lung injury, Ferroptosis

## Abstract

**Purpose:**

Chronic intermittent hypoxia (CIH) causes lung injury but the mechanism is unclear. Ferroptosis is a novel form of programmed cell death. In this research, we attempted to explore the role of ferroptosis in CIH-induced lung injury both in vitro and in vivo.

**Methods:**

Sprague-Dawley rats were randomly separated into control group, CIH group and CIH + ferrostatin-1 group (CIH + Fer-1). Rats in the CIH group and CIH + Fer-1 group were exposed to intermittent hypoxia for 12 weeks. Human bronchial epithelial cell line (BEAS-2B) was cultivated for 24 h in either conventional culture medium or under CIH conditions. Fer-1 was applied to observe its treatment effects. Histological changes were evaluated by Hematoxylin–eosin (HE) staining and masson staining. The expression levels of Acyl-CoA synthetase long-chain family member 4 (ACSL4), glutathione peroxidase 4 (GPX4), interleukin-6 (IL-6) and tumour necrosis factor α (TNFα) were detected via qRT-PCR or Western blot. Cell counting kit-8 (CCK-8) was used to assess cell viability. The apoptotic rate and reactive oxygen species (ROS) was calculated by flow cytometry.

**Results:**

Histology showed that CIH treatment induced lung injury and pulmonary fibrosis in lung tissue. After Fer-1 treatment, the pathological changes caused by CIH alleviated. The mRNA and protein levels of GPX4 decreased significantly in lung tissues of CIH-treated rats and BEAS-2B, (*p* < 0.05). The mRNA and protein levels of ACSL4 increased significantly in lung tissues of CIH-treated rats and BEAS-2B, (*p* < 0.05). The mRNA levels of IL-6 and TNFα in BEAS-2B increased after CIH treatment, (*p* < 0.05). Cell viability decreased, apoptosis rate and ROS increased in CIH-treated BEAS-2B, (*p* < 0.05). Cotreatment with Fer-1 reversed CIH-induced apoptosis, cell viability, ROS accumulation, mRNA and protein levels of GPX4, ACSL4, IL-6 and TNFα both in vitro and in vivo (*p* < 0.05).

**Conclusions:**

Ferroptosis occurred in CIH-induced lung injury, both in vitro and in vivo. The ferroptosis inhibitor Fer-1 alleviated cell injury and ferroptosis in CIH-treated BEAS-2B and lung tissues of rats.

**Supplementary Information:**

The online version contains supplementary material available at 10.1186/s12890-022-02262-x.

## Introduction

Obstructive sleep apnea (OSA) is a chronic respiratory disease with high incidence. The prevalence of OSA has reached 20% in men and 10% in women both in Western countries and Eastern countries [1]. The pathophysiological characteristics of OSA include chronic intermittent hypoxia (CIH) induced by periodic upper airway collapse or obstruction [1]. Target organ injury in OSA patients has received widespread attention. Cardiovascular injury, insulin resistance, cerebral vascular diseases, kidney injury and non-alcoholic fatty liver disease are common complications of OSA [2–6]. At present, clinical evidences and animal experiments have confirmed that CIH induces lung injury and aggravates existing lung injury [7–10].

In a cohort of 5108 patients followed for 10 years, FEV_1_ and FVC declined more rapidly in patients with high OSA probability compared with low OSA probability [11]. The prevalence of chronic cough, asthma, chronic obstructive pulmonary disease (COPD), lung hypertension and other respiratory diseases in OSA patients was significantly increased [12, 13]. Naranjo et al. [14] reported that hospitalized COPD patients with unrecognized OSA had higher readmission and mortality rates than those without OSA. The above clinical studies suggested that OSA was a risk factor of lung injury. Previous animal studies found that eight weeks of CIH exposure induced inflammation and injury in rat lung tissue [7]. Twelve weeks of CIH exposure caused ROS aggregation, mitochondrial damage and apoptosis in rat lung tissue [8]. The molecular mechanism by which CIH causes and aggravates lung injury is, however, unclear.

Ferroptosis is a common type of cell death which is iron-regulated, characterized by lipid-peroxidation [15]. Ferroptosis has been extensively studied in recent years and is involved in the development of various ischemia-reperfusion diseases [16]. The pathophysiological characteristics of CIH are similar to ischemia-reperfusion injury. Furthermore, Chen et al. [17] found that CIH exposure induced ferroptosis in rat liver tissue. However, there was no literature to report whether ferroptosis regulated CIH-related lung injury.

This study explored the role of ferroptosis in CIH-related lung injury using cell and animal experiments. Meanwhile, the treatment effectiveness of ferroptosis inhibitor ferrostatin-1 (Fer-1) was observed both in vitro and in vivo.

## Materials and methods

### Experimental animals and subgroups

The study included male Sprague-Dawley rats aged 8 weeks. The rats were purchased from the Chinese Academy of Sciences' Animal Center (Shanghai, China). All rats were kept in an animal facility with a 12-h light/dark cycle, regular food and tap water. The rats were separated into control group (n = 6), CIH group (n = 6) and CIH + Fer-1 group (n = 6) via computer random number method. The rats in the CIH group and CIH + Fer-1 group were put into a computer-controlled intermittent low oxygen tank. Nitrogen was admitted to the intermittent low oxygen tank for 40 s to decrease the fractional oxygen concentration (FcO_2_) to 6%, stabilized at that level for 20 s, after which oxygen was introduced for the next 40 s to increase FcO_2_ to 21% and maintained in that state for 20 s. This intermittent low oxygen cycle was maintained for 8 h each day for 12 weeks in CIH group and CIH + Fer-1 group [7, 18, 19]. After 8 weeks of CIH treatment, the CIH + Fer-1 group were given 2 mg/kg Fer-1 via abdominal injection. After modeling, the rats were intraperitoneally injected with 50 mg/kg pentobarbital sodium, and lung tissues were harvested for the histopathological examination, masson staining, Western blot analysis and measurement of genetic expression. The Ethics Committee of the Fujian Medical University Laboratory Animal Center approved this study (approve number: IACUCFJMU2022-0031), and all rats were euthanized in accordance with ARRIVE guidelines and treated humanely.

### Cell culture

Human bronchoalveolar epithelial cells (BEAS-2B), provided by Biyuntian Biological Technology Co., Ltd (Shanghai, China). The cell lines were commercial cell lines derived from normal human bronchial epithelial tissue. BEAS-2B were cultured in an incubator at 37 °C with 5% CO_2_. The culture medium was a mixture of DMEM (HyClone Laboratory INC., USA), 10% (vol/vol) fetal bovine serum (Gibco Life Technologies INC., USA) with penicillin (100 units/mL) and streptomycin (100 µg/mL). BEAS-2B were divided into control group, CIH group, and CIH + Fer-1 group. For the CIH group, once BEAS-2B cells reached around 75% confluency, CIH exposure was applied for 24 h by cycling between hypoxia (1% O_2_ with 5% CO_2_ balanced with N_2_ for 60 min) and normoxia (21% O_2_ with 5% CO_2_ balanced with N_2_ for 30 min). For the CIH + Fer-1 group, BEAS-2B was co-treated with 10 µM Fer-1 and CIH for 24 h.

### Hematoxylin–eosin (HE) staining and masson staining of lung tissue of rats

The lung tissue was harvested and immersion-fixed in 4% polymethanol for 24 h, dehydrated in gradient ethanol and then handled to get paraffin blocks. The lung samples were sectioned into 4 µm thicknesses, stained with hematoxylin and eosin or masson trichrome, then observed under a light microscope (Olympus BX50, Tokyo, Japan).

### Protein extraction and Western blot analysis

The harvested lung tissue and BEAS-2B were used to extract proteins using Radio Immunoprecipitation Assay (RIPA) lysis buffer (Beyotime, China). The protein concentration was determined by BCA protein concentration kit (Beyotime, China). Sodium dodecyl sulfate-polyacrylamide gel electrophoresis (SDS-PAGE) was done in advance to separate the proteins. The proteins were subsequently electroblotted onto polyvinylidene difluoride membranes (Millipore, 150 Billerica, MA, USA). The membranes were blocked for 2 h in a solution of 5% nonfat dry milk in Tris Buffer Solution Tween (TBST). The primary antibodies ACSL4 (Acyl-CoA synthetase long-chain family member 4, Abcam, 1: 1000), GPX4 (glutathione peroxidase 4, Abcam, 1: 1000) and β-actin (Abcam, 1: 1000) were then incubated at 4 °C overnight. The membrane was washed with TBST every 10 min. Membranes were washed and stained for 1 h at room temperature with goat anti-rabbit IgG secondary antibody (Abcam, 1: 10,000). After that, the membranes were washed three times with TBST every 10 min. The blots were cut prior to hybridisation with antibodies during blotting. The bands were developed using an improved ECL kit (Thermo Scientific, Rockford, USA). GelDoc XR equipment was used to take the images, which were then analyzed using Image Lab Software (Bio-Rad). Additional file 1 is the original WB image in the manuscript.

### RNA extraction and quantitative reverse transcription polymerase chain reaction (qRT-PCR)

For RNA extraction, ground lung tissue and BEAS-2B were utilized. Total RNA was extracted using the Total RNA Isolation Kit V2 (Vazyme, China), and cDNA was produced using the HiScript III All-in-one RT SuperMix Perfect for qPCR (Vazyme, China). RT-qPCR was performed on an ABI 7500 thermocycler (Applied Biosystems, Foster City, CA, USA). The mRNA expression levels of GPX4 and ACSL4 were assessed using the SYBR Green PCR Master Mix (Vazyme). The suitable primers were summarized in Table 1. The relative expression levels of mRNA were reported as fold change compared to levels detected in controls via the ΔΔCt method.

**Table 1:** PCR primer sequence

Genes	Primer sequence
Hsa-GPX4	F:CCGCTGTGGAAGTGGATGAAGATC
	R: CTTGTCGATGAGGAACTGTGGAG
Hsa-ACSL4	F: TCTGCTTCTGCTGCCCAATT
Has-IL-6 Has-TNFα	R: CGCCTTCTTGCCAGTCTTTT F: GGTGTTGCCTGCTGCCTTCC R: GTTCTGAAGAGGTGAGTGGCTGTC F: TGGCGTGGAGCTGAGAGATAACC R: CGATGCGGCTGATGGTGTGG
Hsa-β-ACTIN	F: TGGCACCCAGCACAATGAA
	R: CTAAGTCATAGTCCGCCTAGAAGCA
Rat-GPX4	F: GGCAGGAGCCAGGAAGTAAT
	R: TGGGCATCGTCCCCATTTAC
Rat-ACSL4	F: GACAGAATCATGCGGTGCTG
	R: TAACCACCTTCCTGCCAGTC
Rat-β-ACTIN	F: CGCGAGTACAACCTTCTTGC
	R: CCTTCTGACCCATACCCACC

### Cell viability test

The viability of BEAS-2B cells was determined using a CCK-8 kit (Beyotime Bio Inc., China). A 96-well plate was seeded with 10,000 cells per well, with five replicate wells in each group. To minimize errors, phosphate-buffered saline (PBS) was added to the surrounding wells. After modeling, cells were incubated for 2 h at 37 °C in fresh media containing 1 mg/mL CCK-8 solution. The absorbance at 450 nm was measured using a microplate reader. Cell viability was calculated as (experimental group-blank control)/ (negative control-blank control) × 100%.

### Apoptosis assay

The apoptosis rate was detected by flow cytometry. 1 × 10^5^ cells were collected per well after modeling, with three replicate wells in each group. Cells were resuspended in 200 µl binding buffer with 10 µl PI and 5 µl Annexin V-FITC after being washed with PBS. A C6 flow cytometer was used to examine the cell apoptosis rate (Becton Dickinson, USA).

### Statistical analysis

The mean standard deviation was utilized when the data were normally distributed and the independent t-test was used to compare the means of two independent groups. A *p*-value of less than 0.05 was considered statistically significant. Graphpad Prism 7.0 was used to conduct the statistical analyses (GraphPad Software Inc., USA).

## Results

### Histopathological changes in CIH-treated lung tissue of rats

**Fig. 1:**
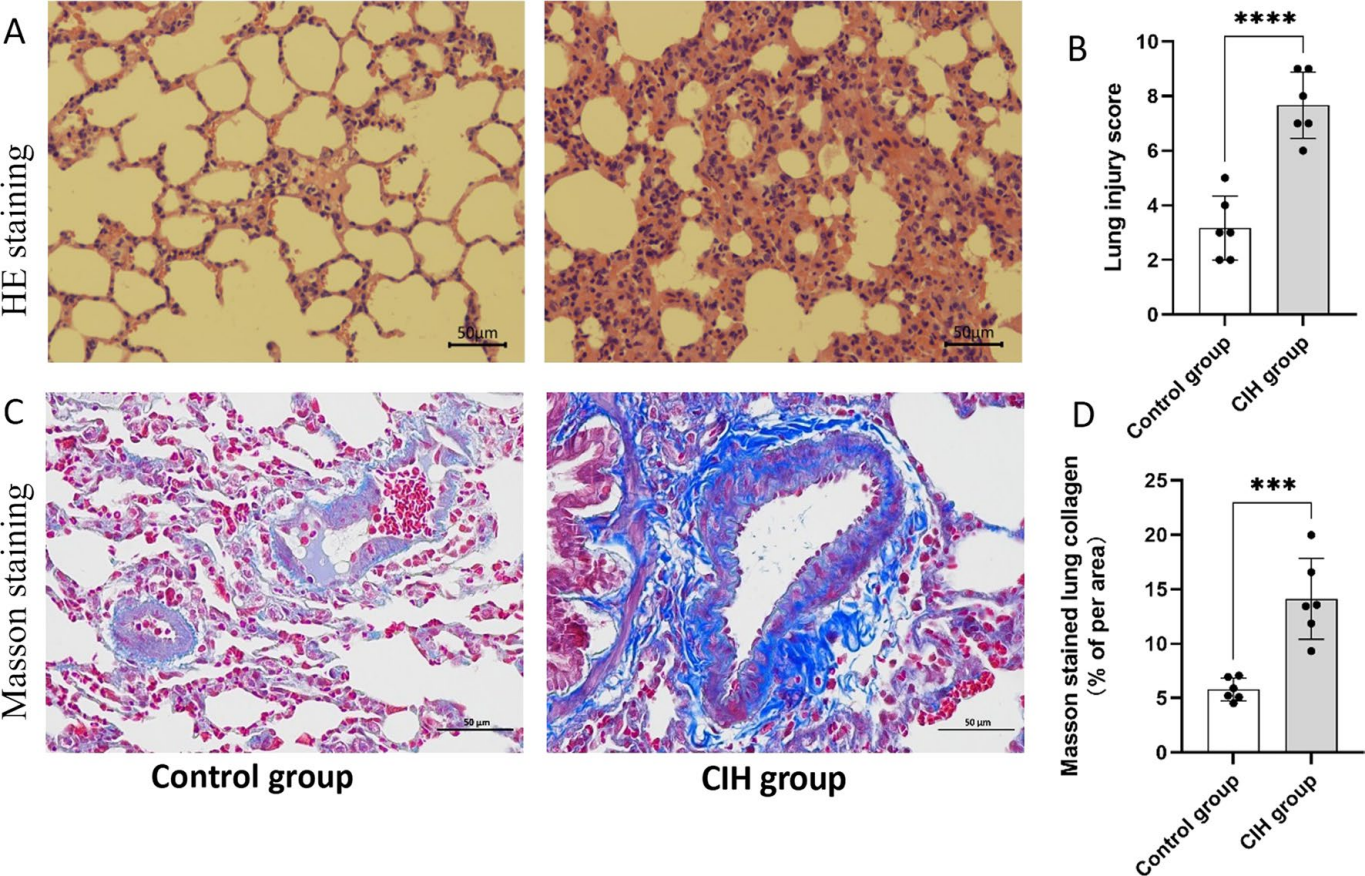
Histopathological alterations in lung tissue of rats after CIH treatment (n = 6). **A** HE staining of lung tissue in control group and CIH group; **B** Quantitative analysis of lung injury score based on HE staining; **C** Masson staining of lung tissue in control group and CIH group; **D** The proportion of collagen areas in lung tissue based on masson staining in control group and CIH group. Data are shown as the mean ± SD. ***p < 0. 001; ****p < 0. 0001

To observe whether CIH injured the lung architecture, HE staining and masson staining histopathological analyses of the lung tissue were performed. The lung tissue in the CIH group exhibited elevated lymphocyte and granulocyte infiltration compared with the control group. Furthermore, the CIH group had increased bleeding and edema in the alveolar compartment and increased thickness of the alveolar wall and vascular wall (Fig. 1A). Lung injury scores of CIH rats were significantly higher than those of control rats (*p* < 0.05) (Fig. 1B). Masson staining showed that CIH treatment increased the ratio of collagen (both *p* < 0.05) (Fig. 1C, D).

### Ferroptosis in CIH-treated lung tissue of rats

**Fig. 2:**
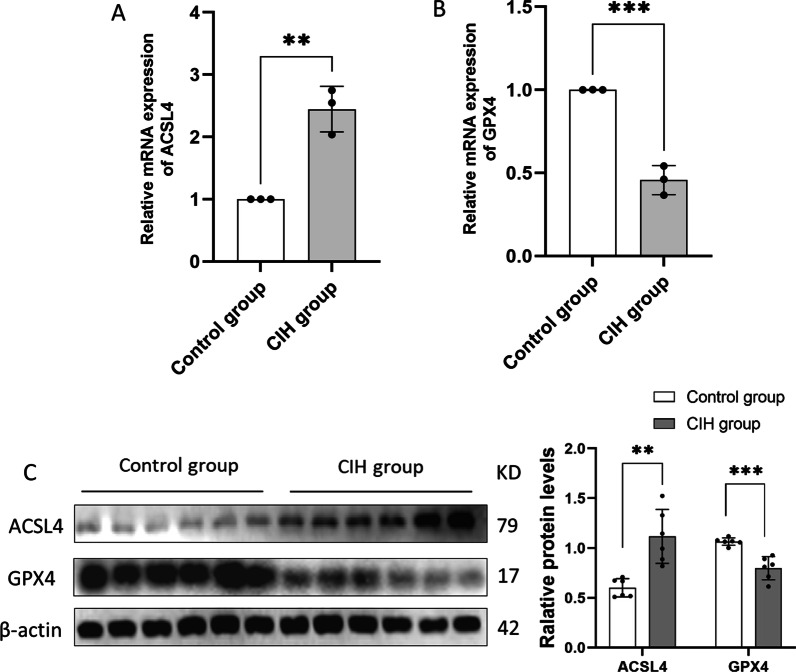
The mRNA and protein levels of GPX4 and ACSL4 in CIH-treated lung tissue of rats (n = 6). **A** CIH increased the mRNA levels of ACSL4;** B**, CIH decreased the mRNA levels of GPX4;** C** The protein levels of ACSL4 and GPX4 in lung tissue of CIH-treated rats. Data are shown as the mean ± SD. **p < 0. 01; ***p < 0. 001

The expression levels of GPX4 and ACSL4 were evaluated to verify ferroptosis in our study. Compared with the control group, ACSL4 mRNA levels were significantly up-regulated (Fig. 2A), while GPX4 mRNA levels were significantly down-regulated in the CIH group (Fig. 2B) (both *p* < 0.05). Simultaneously, ACSL4 protein levels were up-regulated and GPX4 protein levels were down-regulated in the CIH group (both *p* < 0.05), as shown in Fig. 2C.

### CIH-induced BEAS-2B injury and ferroptosis

**Fig. 3:**
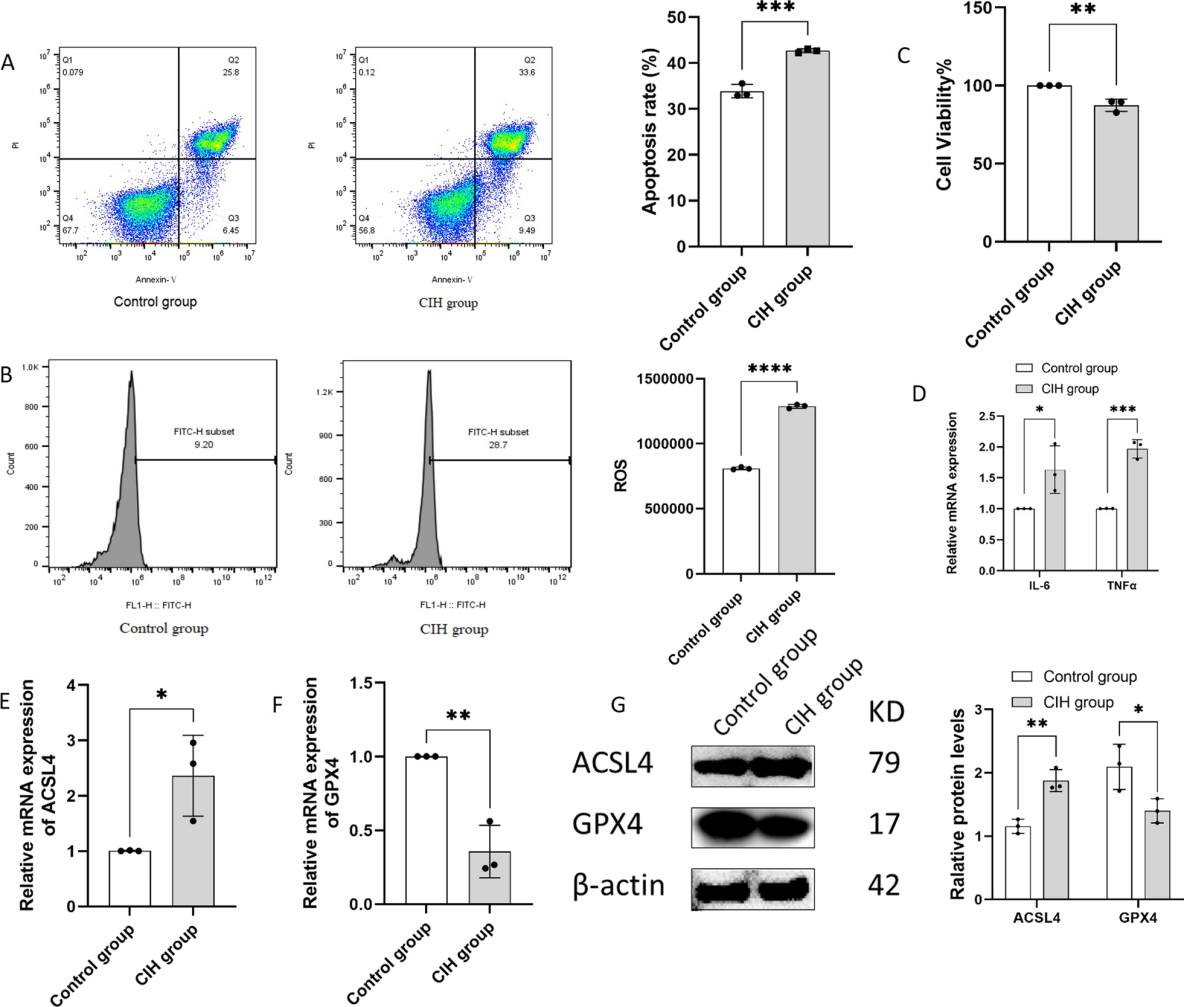
CIH-induced BEAS-2B injury and ferroptosis (n = 3). **A** CIH increased the apoptosis rate of BEAS-2B;** B** CIH increased the ROS levels of BEAS-2B; **C** CIH reduced cell viability of BEAS-2B; **D** CIH increased the mRNA levels of IL-6 and TNFα; **E** the mRNA levels of ACSL4 in Control group and CIH group; **F** the mRNA levels of GPX4 in Control group and CIH group; **G** The protein levels of ACSL4 and GPX4 in Control group and CIH group. Data are shown as the mean ± SD. *p < 0.05; **p < 0.01; ***p < 0.001; ****p < 0.0001

The cell viability of BEAS-2B reduced significantly after 24 h of intermittent hypoxia compared with the control group (100% ± 0% vs. 87% ± 4%, *p* < 0.05) (Fig. 3C). In the CIH group, the apoptotic rate of BEAS-2B increased greatly when compared with the control group (33.9% ± 1. 2% vs. 42.7% ± 0.3%, *p* < 0.05) (Fig. 3A). The levels of ROS were significantly elevated in the CIH group compared with the control group (*p* < 0.05) (Fig. 3B). The levels of IL-6 and TNFα mRNA were increased in CIH group compared with control group (both *p* < 0.05) (Fig. 3D). The mRNA and protein levels of ACSL4 were significantly higher in the CIH group than in the control group, while the mRNA and protein levels of GPX4 were significantly lower, all *p* < 0.05, as shown in Fig. 3E–G.

### Fer-1 alleviated CIH-related BEAS-2B injury and ferroptosis

**Fig. 4:**
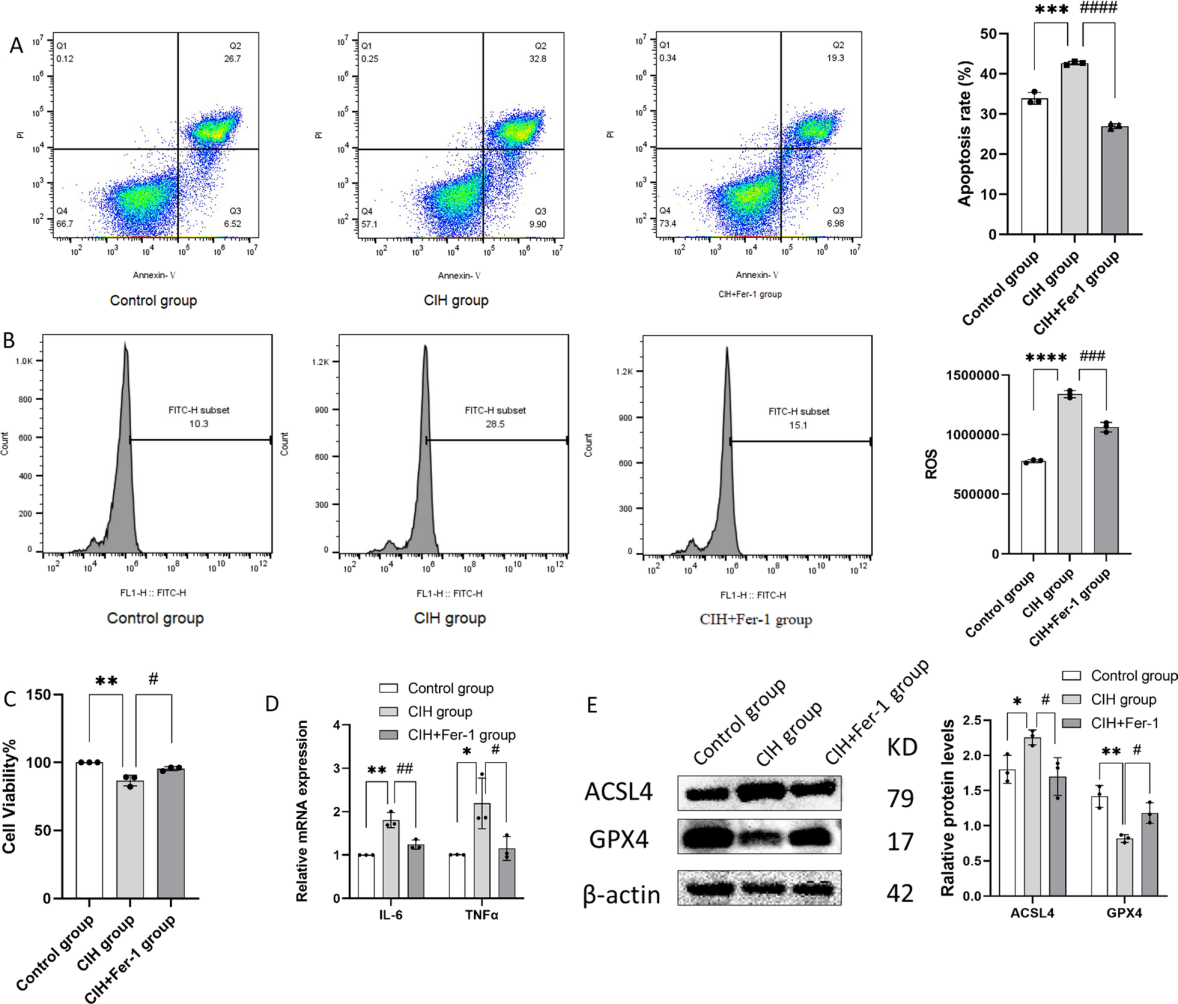
Effects of Fer-1 on CIH-treated BEAS-2B (n = 3). **A** Fer-1 mitigated cellular apoptosis induced by CIH; **B** Fer-1 reversed the ROS accumulation induced by CIH; **C** Fer-1 improved the cell viability of CIH treated BEAS-2B; **D** Fer-1 reduced the mRNA expression levels of IL-6 and TNFα in CIH treated BEAS-2B; **E** the protein levels of GPX4 and ACSL4 in control group, CIH group and CIH + Fer-1 group of BEAS-2B. Data are shown as the mean ± SD. Compared with the control group, *p < 0.05, **p < 0.01, ***p < 0.001, ****p < 0.0001; Compared with the CIH group, #*p* < 0.05, ##*p* < 0.01, ###*p* < 0.001, ####*p* < 0.0001

Cell viability improved (Fig. 4C), apoptosis rate decreased (Fig. 4A) and ROS accumulation decreased (Fig. 4B) in BEAS-2B co-treated with CIH and Fer-1 compared with the CIH group (all *p* < 0.05). In the CIH + Fer-1 group, the protein levels of ACSL4 decreased and the protein levels of GPX4 increased compared with the CIH group (both *p* < 0.05) (Fig. 4E), meanwhile the mRNA levels of IL-6 and TNFα decreased (both *p* < 0.05) (Fig. 4D).

### Fer-1 alleviated CIH related rats lung tissue injury and ferroptosis

**Fig. 5:**
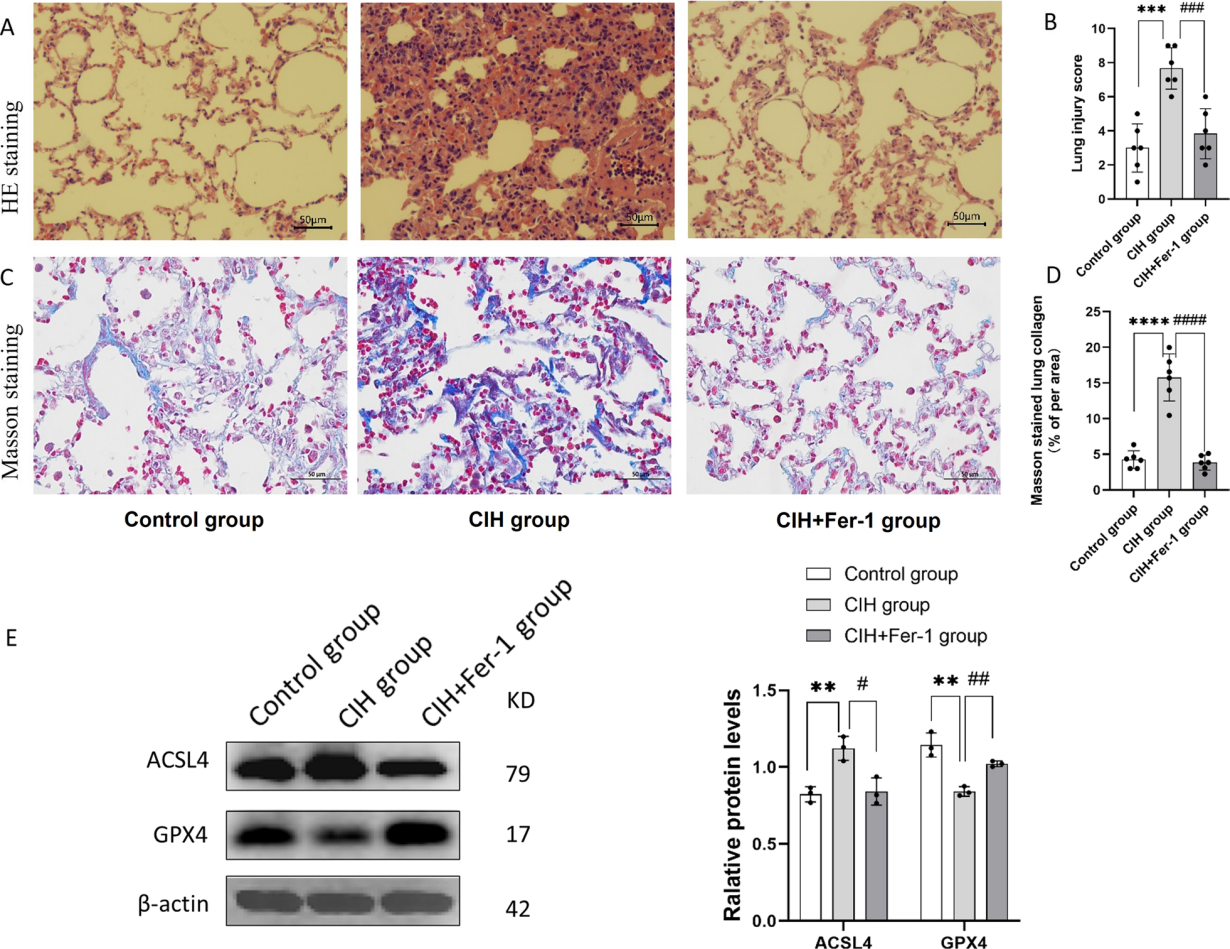
Effects of Fer-1 on CIH-treated lung tissue of rats (n = 6). **A** HE staining of lung tissue in control group, CIH group and CIH + Fer-1 group; **B** Quantitative analysis of lung injury scores based on HE staining; **C** Masson staining of lung tissue in control group, CIH group and CIH + Fer-1 group; **D** The proportion of collagen areas in lung tissue based on masson staining.** E** the protein levels of GPX4 and ACSL4 in control group, CIH group and CIH + Fer-1 group in lung tissue of rats. Data are shown as the mean ± SD. Compared with the control group, *p < 0.05, **p < 0.01, ***p < 0.001, ****p < 0.0001; Compared with the CIH group, #*p* < 0.05, ##*p* < 0.01, ###*p* < 0.001, ####*p* < 0.0001

We further conducted animal experiments to verify whether Fer-1 reversed CIH-induced lung injury. The results of HE staining showed that lung injury scores decreased after Fer-1 treatment (*p* < 0.05) (Fig. 5A, B). The results of masson staining showed that Fer-1 treatment attenuated the ratio of collagen caused by CIH (both *p* < 0.05) (Fig. 5C, D). The results of Western blot showed that Fer-1 treatment reversed the protein expression levels of ACSL4 and GPX4 (both *p* < 0.05) (Fig. 5E).

## Discussion

The current investigation demonstrated that CIH exposure increased the advancement of ferroptosis in bronchoalveolar epithelial cells and lung tissue. The intermittent hypoxia-related bronchoalveolar epithelial cell injury can be effectively inhibited by ferroptosis inhibitor Fer-1. This study provided a novel insight into CIH-induced lung injury.

Previous clinical studies found that OSA caused and aggravated various pulmonary diseases [20]. In addition, several prior animal investigations have verified lung injury caused by CIH [7, 21–23]. Inflammatory reactions and cellular apoptosis of lung tissue after intermittent hypoxia were the focus of previous studies [24, 25]. Ferroptosis is a novel type of cell death, which is different from apoptosis and necrosis [26]. Less attention has been focused on CIH-related ferroptosis in lung tissue. In this study, we established a CIH rat model to simulate the OSA hypoxic model. Besides histological lung tissue injury, we found ferroptosis-related protein dysregulation in the lung tissue of CIH-treated rats. Overexpression or knockdown of GPX4 regulated the cell mortality after various ferroptosis inducers treatment, so GPX4 has been identified as a key regulator of ferroptosis [27]. ACSL4 was identified as a key enzyme regulating lipid composition and promoting ferroptosis [28]. The decrease in GPX4 and the increase in ACSL4 have been utilized as indicators of ferroptosis. The dysregulation of GPX4 and ACSL4 in vitro and in vivo in this study confirmed the occurrence of ferroptosis after CIH treatment.

**Fig. 6:**
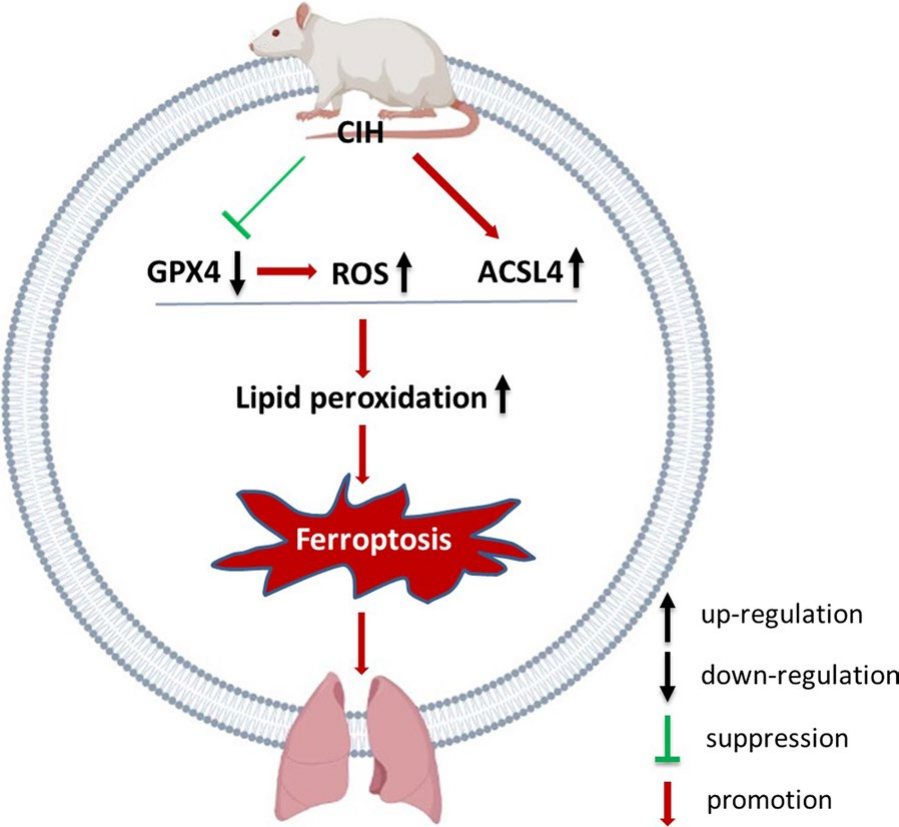
Proposed mechanisms of bronchoalveolar epithelial cells ferroptosis induced by CIH. CIH inhibited GPX4 activity which produced a lot of ROS, activated ACSL4, as a result promoted lipid peroxidation, and induced ferroptosis, eventually caused lung injury

Ferroptosis was involved in the development and progression of a variety of lung diseases, including acute lung injury, COPD, and asthma [29–31]. Yoshida et al. [32] investigated the involvement of ferroptosis in the development and progression of COPD both in vitro and in vivo. Han et al. [33] found that IL-6 promoted ferroptosis in bronchial epithelial cells by inducing ROS-dependent lipid peroxidation. The injury induced by hypoxia and reoxygenation during CIH was similar to that induced by ischemia-reperfusion injury. Li et al. found that ferroptosis regulated intestinal ischemia/ reperfusion-induced acute lung injury via P53 [16]. We speculated the CIH-related ferroptosis in our study may also be associated with hypoxia and reoxygenation injury. Frequent cycles of hypoxia and reoxygenation resulted in the production of ROS, which triggered lipid peroxidation and ultimately ferroptosis [28]. In this study, the significant accumulation of ROS in CIH-treated BEAS-2B was consistent with our prediction. A graphic summary was shown in Fig. 6.

One of the most well-known ferroptosis inhibitors is Fer-1 [34], which has been widely used in in vitro and in vivo studies. A previous study confirmed that Fer-1 alleviated cigarette smoke extract-induced BEAS-2B injury [35]. In addition, Fer-1 inhibited ferroptosis and alleviated lipopolysaccharide-induced acute lung injury [35]. Similar to previous studies, Fer-1 decreased the apoptosis rate and improved cell viability in CIH-treated BEAS-2B, and it also attenuated the pathological changes of lung tissue in CIH treated rats, which suggested the therapeutic potential of Fer-1 in CIH-related lung injury. Fer-1 usually ameliorated ferroptosis via inhibiting lipid peroxidation [36]. In the study, the ROS levels reduced after Fer-1 treatment, which confirmed Fer-1 alleviating oxidative stress in CIH-treated BEAS-2B. The mRNA levels of IL-6 and TNFα decreased after Fer-1 treatment, which indicated that Fer-1 reversed the inflammation caused by CIH exposure. In this study, the expression levels of GPX4 increased and the expression levels of ACSL4 decreased both in CIH-treated BEAS-2B and lung tissues of CIH-treated rats after treatment with Fer-1. These findings also showed Fer-1 reversed ferroptosis in CIH-treated BEAS-2B and lung tissue.

In addition to Fer-1, other types of ferroptosis inhibitors, lipoxstatin-1 and IASPP (an inhibitor of p53 transcriptional activity), were found to alleviate the ischemia-reperfusion-induced acute lung injury via inhibiting ferroptosis [37, 38]. The pathophysiological mechanisms of intermittent hypoxia and ischemia-reperfusion were similar. So the role of these ferroptosis inhibitors in CIH-related lung injury should be explored in future studies.

However, there are some limitations to this study. First, due to financial constraints, the sample size of animal experiments was relatively small. Second, although this study confirmed the occurrence of ferroptosis and the efficacy of Fer-1 in treatment of CIH-related lung injury, the mechanism was not explored in depth.

In conclusion, our results revealed that ferroptosis was involved in CIH-induced lung injury both in vitro and in vivo. Fer-1, a ferroptosis inhibitor, reversed the CIH-related lung injury.

## Supplementary Information

**Additional file 1**. Original Western blot images in the manusctipt.

## Data Availability

The data used in this study can be obtained through the corresponding author.

## Abbreviations

CIH: Chronic intermittent hypoxia
Fer-1: Ferrostatin-1
BEAS-2B: Human bronchial epithelial cell line
HE staining: Hematoxylin–eosin staining
ACSL4: Acyl-CoA synthetase long-chain family member 4
GPX4: Glutathione peroxidase 4
IL-6: Interleukin-6
TNFα: Tumor necrosis factor α
CCK-8: Cell counting kit-8
ROS: Reactive oxygen species
OSA: Obstructive sleep apnea
COPD: Chronic obstructive pulmonary disease
RIPA: Radio immunoprecipitation assay
SDS-PAGE: Sodium dodecyl sulfate-polyacrylamide gel electrophoresis
TBST: Tris buffer solution tween
PBS: Phosphate-buffered saline
