# Supplementary material for: The role of ferroptosis in chronic intermittent hypoxia-induced lung injury
Source: BMC Pulm Med. 2022 Dec 27;22:488. doi: 10.1186/s12890-022-02262-x (PMC9793575; doi:10.1186/s12890-022-02262-x)
Supplement: Supplementary file 1 — Additional file 1. Original Western blot images in the manusctipt. [file 12890_2022_2262_MOESM1_ESM.pdf]

Supplementary Material

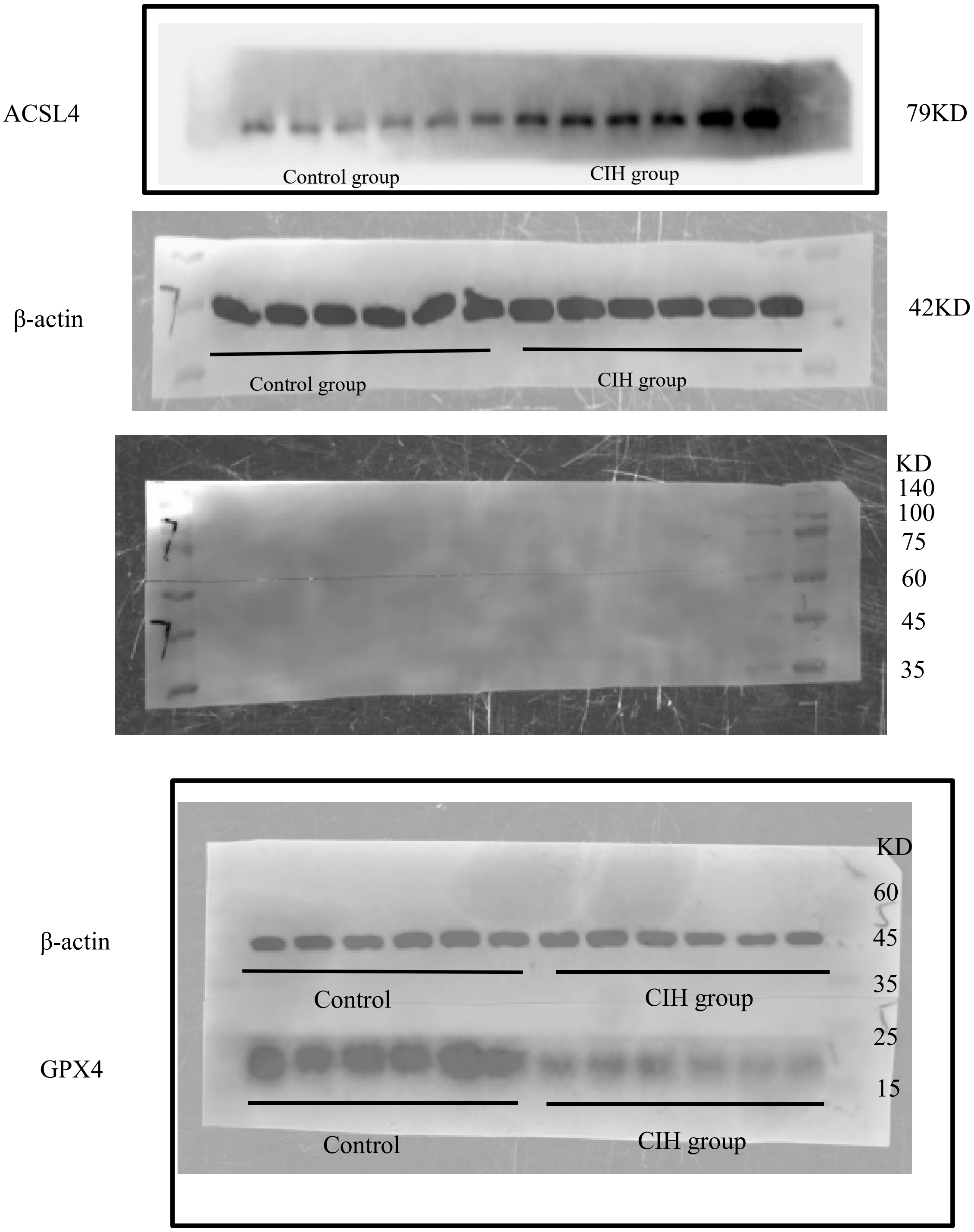

Figure S1. Western blot images of Fig. 2C. The WB legends of the rats' lung tissue. The blots were cut prior to hybridisation with antibodies during blotting. The images show all blots and replicates of ACSL4, GPX4, and β-actin. The inner black rectangle are the representative blots.

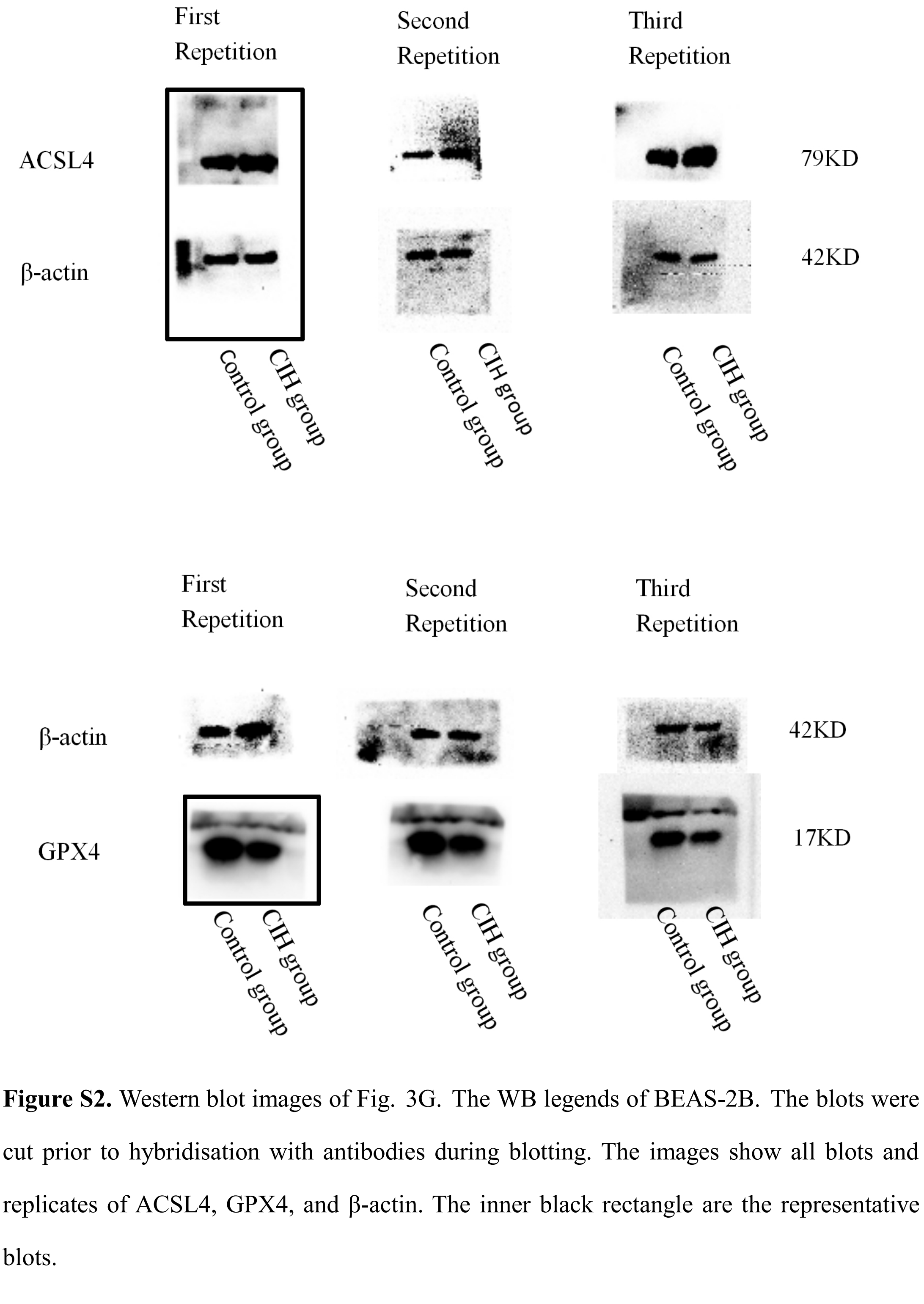

Figure S2. Western blot images of Fig. 3G. The WB legends of BEAS-2B. The blots were cut prior to hybridisation with antibodies during blotting. The images show all blots and replicates of ACSL4, GPX4, and β-actin. The inner black rectangle are the representative blots.

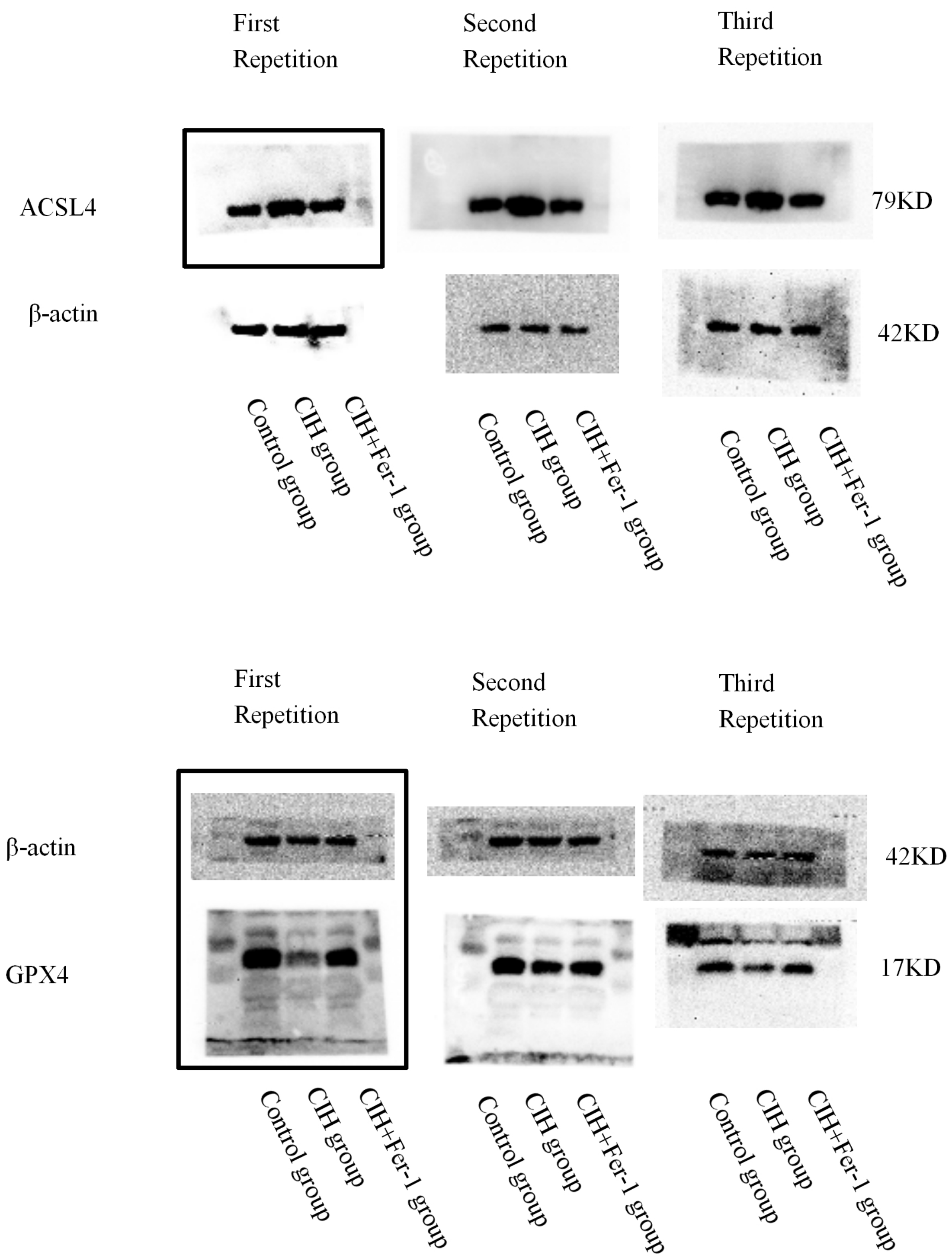

Figure S3. Western blot images of Fig. 4E. The WB legends of BEAS-2B. The blots were cut prior to hybridisation with antibodies during blotting. The images show all blots and replicates of ACSL4, GPX4, and β-actin. The inner black rectangle are the representative blots.

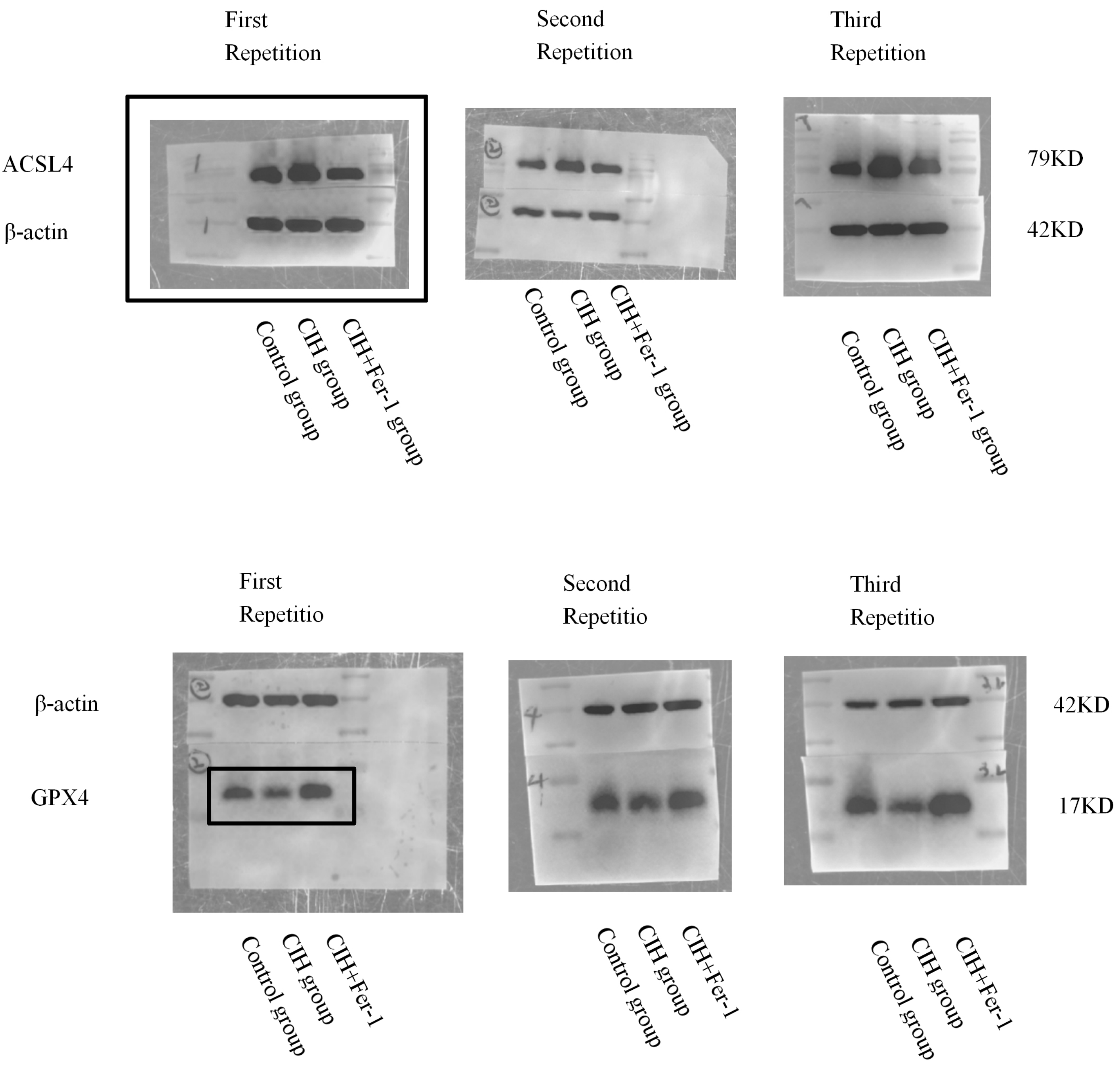

Figure S4. Western blot images of Fig. 5E. The WB legends of the rats′ lung tissue. The blots were cut prior to hybridisation with antibodies during blotting. The images show all blots and replicates of ACSL4, GPX4, and β-actin. The inner black rectangle are the representative blots.
